# Serum Concentrations of Vitamin D, Calcium, Phosphorus and Trace Minerals in Adults and Children with Haemophilia A: Association with Disease Severity, Quality of Life, Joint Health and Functional Status

**Published:** 2020-01-01

**Authors:** Amir Abbasnezhad, Mehdi Habibi, Babak Abdolkarimi, Soodabeh Zare, Ezatollah Fazeli Moghadam, Razieh Choghakhori

**Affiliations:** 1 Nutritional Health Research Center, Lorestan University of Medical Sciences, Khorramabad, Iran; 2 Department of Biostatistics, School of Medical Sciences, Tarbiat Modares University, Tehran, Iran; 3 Razi Herbal Medicines Research Center, Lorestan University of Medical Sciences, Khorramabad, Iran

**Keywords:** Haemophilia, Vitamin D, Pediatric haemophilia/haemophilia activities list, Haemophilia joint health score, Haemophilia-specific quality of life

## Abstract

**Background** **:** To investigate the serum levels of 25(OH)D and minerals in adults and children with haemophilia A, and the possible association of these factors with Pediatric Haemophilia/Haemophilia Activities List (PedHAL/HAL), Haemophilia Joint Health Score (HJHS) and Haemophilia-specific quality of life (QoL) index this case-control study was conducted.

**Materials and Methods:** Eighty five haemophilia A patients (HP) registered in Hemophilia Society of Lorestan province were recruited. Along with HP, sex and age matched healthy controls (HCs) were recruited. Linear regression was used to evaluate the possible relation between biochemical factors and other variables. One-way analysis of variance (ANOVA) was used to compare the biochemical factors between three or more independent groups.

**Results:** Results indicated that serum zinc, phosphorus and magnesium were significantly lower, whereas, serum level of alkaline phosphatase (ALP) was statistically higher in HP compared with HCs. Other biochemical factors including calcium and parathyroid hormone (PTH) were not different between groups. Serum 25(OH) D was lower only in children with haemophilia and not in adults. Percentage of subjects who were vitamin D deficient was higher in HP vs. HCs (57.6% vs. 35.3%), and also this rate was higher in children with haemophilia vs. adults (77.8% vs. 48.3%). Lower serum concentrations of assessed minerals and vitamin D were associated with lower physical activity, poor QoL and worst joint health, and these associations were stronger in children.

**Conclusion:** Present study indicated that serum levels of vitamin D and minerals were low in HP, and these low levels were associated with poor QoL, lower physical activity and worst joint health.

## Introduction

Haemophilia is a X-linked hereditary coagulation disorder, characterized by deficiency of coagulation factor VIII (haemophilia A) or IX (haemophilia B)^   1 ^. Symptoms include acute hemorrhages into the various organs of the body, including joints and musculoskeletal system^   2 ^. Due to the bleeding episodes, other comorbidities such as arthropathy are common in these patients^   2 ^. The prevalence of hemoarthrosis in patients with haemophilia is about 75%–90%, and the most common involved joints are ankle and knee^   3 ^,^4^. In addition, fear of bleeding leads to reduced mobility and weight-bearing exercise, which are associated with reduced bone mineral accrual^5^. Prolonged immobility as well as co-morbidities such as HCV and HIV are predisposing factors for decreased bone mineral density (BMD), which leads to osteoporosis and related fractures^6^^,^^7^. Evidence suggested that these co-morbidities negatively impact psychosocial status such as self-esteem, which is correlated with depression and anxiety^8^^,^^9^. Lower self-esteem as well as depression and anxiety result in an impaired quality of life (QoL) in hemophilic patients (HP)^8^^,^^10^.

In addition to the mentioned predisposed factors, subclinical deficiencies of 25(OH)D, magnesium (Mg) and zinc (Zn) are associated with low BMD and osteoporosis^11^^,^^12^. A limited number of studies have addressed the role of vitamin D in HP, and the results of these studies regarding the vitamin D status in patients with haemophilia are inconsistent. Although, some studies found lower concentrations of 25(OH)D in haemophilia patients vs. healthy controls (HCs)^13^, others reported no differences^14^. Moreover, very few studies have assessed the serum levels of minerals in HP, and most of the finding are controversial^15^^,^^16^. 

As yet, there is little data in the literature addressing the status of vitamin D, minerals and their correlation with clinical and psychological factors in patients with haemophilia, and available studies are limited with the low number of patients. Therefore, we aimed to assess the serum 25(OH)D, Zn, phosphorus and Mg in these patients and compare it with healthy subjects. Furthermore, we aimed to evaluate the possible correlation of vitamin D, Zn, Mg, phosphorus and calcium (Ca) with QoL, joint health and functional status of HP. To our best knowledge, present study is the first study assessing the correlation of vitamin D and minerals with the QoL and functional status in patients with haemophilia. 

## MATERIALS AND METHODS

**Study design and participants**

In this case-control study, all the haemophilia A patients registered till September 2017 in Hemophilia Society of Lorestan province from all the ages were recruited. Haemophilia A patients were diagnosed according to the deficiency of coagulation factor VIII and classified according to the activity of factor VIII as mild >5 units/dL, moderate 1–5 unit/dL, and severe <1 unit/dL according to Arnold and Hilgartner^17^. Along with haemophilia patients, sex and age matched healthy subjects were recruited from local population via advertisement. Excluding criteria were any evidence of chronic diseases producing osteopenia/osteoporosis, smoking, alcohol consumption, thyroid or parathyroid disorders, history of chronic renal, hepatic, or gastrointestinal disease, use of steroidal anti-inflammatory drugs, antiepileptic medication or any drug that can affect bone metabolism. Furthermore, additional exclusion criteria were being on a special diet, iron, Ca, Zn, Mg, vitamin D, multivitamin and mineral supplementation over the past six months. Patients with HIV, hepatitis B and HCV were also excluded. After allocation, the participants filled in the questionnaires, and went to the lab for blood sampling the next day. Socio-demographic characteristics and medical history of all the participants were extracted from the general questionnaire through face-to-face interviews. The weight was measured to the nearest 100 g, while the participants were minimally clothed and were without shoes. Height was measured to the nearest 0.1 cm without shoes with shoulders in a normal position. Then, BMI was calculated. The study protocol was approved by the Medical Ethics Committee at the Lorestan University of Medical Sciences (Registration No. LUMS.REC.1396.263), and was conducted in accordance with the International Conference on Harmonization Good Clinical Practice guidelines and the Declaration of Helsinki. The study protocol was fully explained to the participants, and the written informed consent was obtained from all the participants.

**Laboratory analysis**

After 8-12 hours overnight fasting, five milliliters of blood samples were drawn from each subject. After the centrifugation of the blood samples, the serums were frozen at −80 °C until laboratory analyses were done. For evaluating vitamin D status, serum 25(OH)D_3_ levels were measured using radioimmunoassay method (Immunodiagnostic Systems, Boldon, UK), and serum Ca, Mg and phosphorus were measured using a photometric test (Pars Azmoon Co, Tehran, Iran). The serum level of Zn was measured by using the flawless atomic absorption spectrometry (Perkin-Elmer Corp., Norwalk, Conn. Germany). The enzyme-linked immunosorbent assay (ELISA) method was used to measure serum levels of alkaline phosphatase (ALP) (MicroVue BSAP; Quidel Corporation, San Diego, CA, USA), and parathyroid hormone (PTH) (Immunodiagnostic System, Fontain Hills, AZ). All the laboratory analyzes were done in the Central Laboratory at the Lorestan University of Medical Sciences, and all the technicians were blind to the case and control status.

**Haemophilia Joint Health Score (HJHS)**

To evaluate joint impairment, new joint scoring instrument the Haemophilia Joint Health Score (HJHS) version 2.1 was used^18^. This instrument comprised items including: swelling, duration of swelling, muscular atrophy, crepitus on motion, loss of range of motion in extension and flexion, joint pain, strength, and global gait, and assesses the early signs of arthropathy in six main joints of the elbows, knees and ankles. The HJHS total score can be calculated by summing the joint total scores with the global gait score, which 0 represent the perfect and 124 the worst joint health.

**Haemophilia-specific quality of life index (Haemo-QoL and Haem-A-QoL questionnaires)**

The Haem-A-QoL instrument is the disease specific questionnaire to assess QoL of adult patients with haemophilia. This instrument has 10 dimensions and 46 items (dimensions: physical health, feelings, view, sport and leisure time, work and school, dealing, treatment, future, family planning, and relationships/partners). Score of each dimension as well as the total score is transformed to a scale from 0 to 100. Higher scores represent the worse HRQoL.

To assess QoL of children and adolescents with haemophilia, the long version of Haemo-QoL questionnaire was used after approval and permission of the Haemo-QoL Group. The younger children version (age group I: 4–7 years) of the questionnaire has 21 items comprising 8 dimensions (physical health, feelings, view, family, friends, others, sport and school/kindergarten, treatment). The school children version (age group II: 8–12 years) contains 2 additional domains (received support and dealing) with overall 64 items, and the adolescents version (age group III: 13-16/18 years) has 2 further additional domains (relationships, future) with 77 items. In addition to the Haem-A-QoL, the score of each dimension and the total score of Haemo-QoL are transformed to a 0 to 100 scale, and the higher scores indicate the worse HRQoL. 

**Pediatric Haemophilia/Haemophilia Activities List (PedHAL/HAL)**

The Haemophilia Activities List (HAL) is the disease-specific self-assessment questionnaire to measure the activity and participation status of adult patients with haemophilia. The HAL instrument consists of 45 items and 8 domains (lying/siting/kneeling/standing, function of the legs, function of the arms, use of transportation, self-care, household tasks, and leisure activities and sports). Normalized scores range from 0 to 100 (worst functional status and best functional status, respectively).

The Pediatric Haemophilia Activities List (PedHAL) is an adapted version of the HAL instrument, assessing activities limitations in children and teenagers, consists of same dimensions with 53 items. Similar to the HAL, the overall score is on a scale of 0–100, where 0 represents the worst functional status and 100 the best functional status.

**Statistical analyses**

The normality of the data distribution was analyzed by the Kolmogorov–Smirnov test. Parametric data were reported as mean ± standard deviation (SD), and for non-parametric data median (25th, 75th percentiles) was reported. To compare the baseline characteristics and other covariates between cases and HCs, independent *t*-test (parametric data) or Mann–Whitney *U*-test (non-parametric data) was used. Moreover, for categorical variables, chi-squared test, or Fisher’s exact test was used. The Vitamin D status was categorized according to the circulating level of 25(OH)D3 as deficient (<20 ng/mL), insufficient (≥20 to <30 ng/mL) and sufficient (≥30 ng/mL)^19^. To evaluate the possible relation between biochemical factors and other variables the linear regression was used. In addition, to compare the biochemical factors between mild, moderate and severe haemophilia, the One-way analysis of variance (ANOVA) was used. Post-hoc analysis (Tukey’s test) was used to analyze the differences between specific groups. The significance level was considered 0.05. All statistical analyses were done using SPSS version 16 statistical software (SPSS Inc., Chicago, III).

## Results

**Baseline characteristics**

**Figure 1:**
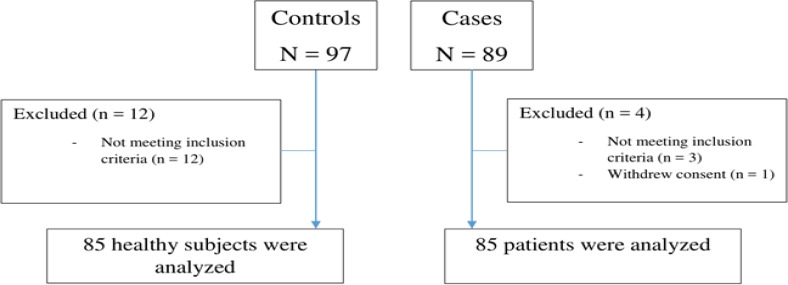
Flowchart of the participants through the study

As Figure 1 demonstrates, of all patients with haemophilia A assessed for the eligibility 4 were excluded because of withdraw consents (n=1) and not meeting inclusion criteria (n=3). In the control group, of 97 healthy subjects, 12 were excluded due to not meeting inclusion criteria. Of 85 haemophilia A patients who were enrolled, 27 were under 18 years old and 58 were equal and above 18 years old. The mean age ± SD was 25.23 ± 10.69 years in HP and 23.31 ± 10.47 years in HCs (Table 1). As table 1 shows, 24.7%, 37.6% and 37.6% of HP had mild, moderate and severe hemophilia, respectively. There were no significant differences (*p*>0.05) in terms of age, weight, BMI, and socio-demographic characteristics between IBS and control groups (Table 1). 

**Serum concentrations of biochemical factors in Hemophilic patients and Healthy controls**

As Table 2 indicates, serum concentrations of Zn, phosphorus and Mg were significantly (*p* <0.05) lower in HP as compared to HCs. Whereas, serum level of ALP was statistically (*p* <0.05) higher in HP. Circulating concentrations of other biochemical factors including 25(OH)D, Ca and PTH were not different between groups (Table 2). When comparing adults separately, only serum levels of Zn and phosphorus were significantly (*p* <0.05) lower, and ALP were higher in haemophilia A compared with HCs. In children with haemophilia A, serum concentrations of 25(OH)D, Zn, phosphorus and Mg were statistically (*p* <0.05) lower as compared with healthy children. In addition, serum ALP was significantly (*p *<0.001) higher in children with haemophilia A (Table 2). Results of the Table 3 shows that the percentage of patients who were vitamin D deficient was higher in hemophilic group compared with healthy group (57.6% vs. 35.3%, respectively). When comparing children and adults separately, the percentage of patients who were vitamin D deficient was higher in both children and adults with haemophilia compared with healthy group (77.8% vs. 44.4%; 48.3% vs. 31%; respectively; Table 3). 

**Table 1:** Subjects characteristics

**Variables**	**Haemophilic patients** **n = 85**	**Healthy ** **subjects** **n = 85**	***p***
Age	25.23 ± 10.69	23.31 ± 10.47	0.236
Severity of Hemophilia, n(%):† Mild Moderate Severe	21 (24.7) 32 (37.6) 32 (37.6)	- - -	-
PedHAL/HAL	51.06 ± 22.13	-	-
HJHS	28.95 ± 14.53	-	-
Haemo-QoL/Haem-A-QoL	40.21 ± 18.71	-	-
Weight	56.12 ± 16.92	60.77 ± 19.57	0.1
BMI	20.36 ± 3.35	21.43 ± 3.76	0.06
Education Level, n (%):† None/ Primary Secondary/High School University or higher	18 (21.2) 37 (43.5) 30 (35.3)	17 (20) 30 (35.3) 38 (44.7)	0.427
Family Income, n (%):† Low income Middle income High income	21 (24.7) 56 (65.9) 8 (9.4)	25 (29.4) 49 (57.6) 11 (12.9)	0.525
Marital status, n (%):† Single Married	64 (75.3) 21 (24.7)	55 (64.7) 30 (35.3)	0.18

All data are shown as mean ± standard deviation, and analyzed by two-sample *t* test unless otherwise indicated.

PedHAL/HAL, Pediatric Haemophilia/Haemophilia Activities List; HJHS, Haemophilia Joint Health Score; Haemo-QoL/Haem-A-QoL, Haemophilia-specific quality of life index.

† Data are numbers (%), and were analyzed by *χ*^2^ test or Fisher’s exact test.

**Table 2:** Comparison of serum concentrations of biochemical factors between total haemophilia A patients and HCs, and between adults and children separately

**Variables**	**Total ** **haemophilia A ** **patients (n=85)**	**HCs** **n = 85**	***p***	**Adults with haemophilia ** **A** **n = 58**	**Healthy adults** **n = 58**	***p***	**Children with ** **haemophilia A** **n = 27**	**Healthy ** **children** **n = 27**	**p **
25(OH)D, ng/mL	21.4 ± 4.08	22.3 ± 3.95	0.147	22.06 ± 4.47	22.38 ± 3.85	0.68	19.97 ± 2.61	22.11 ± 4.21	0.03
Zinc, µgr/dL	64.55 ± 21.5	83.51 ± 26.97	<0.001	65.45 ± 23.4	82.84 ± 25.09	<0.001	62.62 ± 16.95	84.96 ± 31.08	0.002
Phosphorus, mg/dL	3.93 ± 0.66	4.84 ± 2.25	<0.001	4.12 ± 0.61	5.01 ± 2.33	0.006	3.52 ± 0.59	4.48 ± 2.06	0.025
Calcium, mg/dL	8.45 ± 1.18	8.74 ± 0.97	0.09	8.5 ± 1.17	8.82 ± 1.04	0.117	8.36 ± 1.21	8.55 ± 0.78	0.494
Magnesium, mg/dL	1.69 ± 0.88	2.04 ± 0.81	0.007	1.87 ± 0.85	2.17 ± 0.81	0.063	1.29 ± 0.81	1.77 ± 0.76	0.028
ALP, IU/L	237.98 ± 68.33	142.25 ± 65.44	<0.001	211.14 ± 56.12	121.45 ± 52.07	<0.001	295.67 ± 55.82	186.92 ± 69.67	<0.001
PTH, pg/mL	39.81 ± 24.66	34.91 ± 23.14	0.184	35.46 ± 22.29	32.17 ± 21.43	0.419	49.14 ± 27.26	40.82 ± 25.87	0.255

All data are shown as mean ± standard deviation, and analyzed by two-sample *t* test.

ALP, alkaline phosphatase; PTH, parathyroid hormone; HCs, healthy controls.

**Table 3:** Serum 25(OH)D status in Hemophilic patients and HCs.

	**25(OH)D**			**p **
	**< 20 ng/ml**	**≥ 20 to < 30 ng/ml**	**≥ 30 ng/ml**	
Hemophilic patients, n (%)	49 (57.6)	31 (36.5)	5 (5.9)	0.008
HCs, n (%)	30 (35.3)	51 (60)	4 (4.7)	
Adults with haemophilia A, n (%)	28 (48.3)	26 (44.8)	4 (6.9)	0.037
Healthy adults, n (%)	18 (31)	39 (67.2)	1 (1.7)	
Children with haemophilia A, n (%)	21 (77.8)	5 (18.5)	1 (3.7)	0.042
Healthy children, n (%)	12 (44.4)	12 (44.4)	3 (11.1)	

Data are numbers (%), and were analyzed by *χ*^2^ test.

HCs, healthy controls.

**Serum concentrations of biochemical factors according to severity of haemophilia**

Results of the Figure 1 demonstrated the circulating concentrations of biochemical factors according to severity of haemophilia. As figure 1 shows, serum level of the 25(OH)D was significantly lower in severe haemophilia (Tukey’s test:* p* = 0.04) as compared with HCs (severe, moderate, mild vs HCs: 19.74 ± 2.05, 20.67 ± 2.25, 23.21 ± 5.6 vs. 22.3 ± 3.95, respectively; Between groups:* p* <0.001). Serum 

level of Mg was statistically lower in severe haemophilia (Tukey’s test:* p* <0.001) vs. HCs (severe, moderate, mild vs HCs: 1.26 ± 0.66, 1.63 ± 0.77, 2.03 ± 0.98 vs. 2.04 ± 0.81, respectively; between groups:* p* <0.001). Circulating concentration of phosphorus was significantly lower in both moderate and severe haemophilia (Tukey’s test:* p* = 0.04, *p* = 0.02, respectively) when compared with HCs (severe, moderate, mild vs HCs: 3.63 ± 0.64, 3.91 ± 0.65, 4.14 ± 0.63 vs. 4.84 ± 2.25, respectively; between groups:* p* <0.001). Serum level of ALP was statistically higher in mild, moderate and severe haemophilia (Tukey’s test:* p* <0.001, *p* <0.001, *p* <0.001, respectively) as compared with HCs (severe, moderate, mild vs HCs: 274.28 ± 59.38, 245.75 ± 74.57, 203.28 ± 51.91 vs. 142.25 ± 65.44, respectively; between groups:* p* <0.001). Furthermore, serum concentration of Zn was statistically lower in both moderate and severe haemophilia (Tukey’s test:* p* <0.001, *p* <0.001, respectively) vs. HCs (severe, moderate, mild vs HCs: 54.29 ± 9.79, 64.33 ± 9.63, 71.52 ± 31.24 vs. 83.51 ± 26.97, respectively; between groups:* p* <0.001). Serum concentrations of Ca (severe, moderate, mild vs HCs: 8.45 ± 1.34, 8.67 ± 1.32, 8.23 ± 0.87 vs. 8.74 ± 0.97, respectively; between groups: *p* = 0.14) and PTH (severe, moderate, mild vs HCs: 42.42 ± 26.89, 40.03 ± 25.24, 37.86 ± 23.15 vs. 34.91 ± 23.14, respectively; between groups: *p* = 0.53) were not different in various types of severity in HP compared with HCs. 

**Association of serum vitamin D and assessed minerals with Haemo-QoL/Haem-A-QoL**

**Figure 2:**
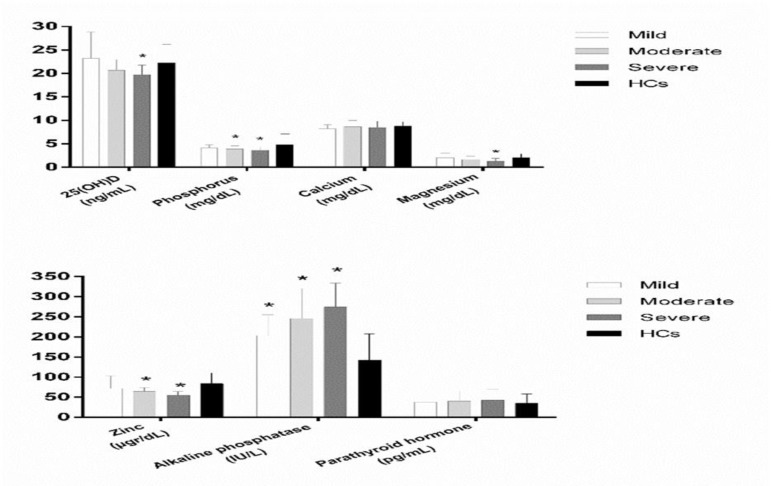
Mean serum levels of 25(OH)D, trace minerals, ALP and PTH in mild, moderate and severe haemophilia in comparison to HCs. (* *p *<0.05). ALP, alkaline phosphatase; PTH, parathyroid hormone; HCs, healthy controls.

According to the Figure 2, serum level of 25(OH)D had a strong negative association with Haemo-QoL/Haem-A-QoL in both children and adults (R = -0.52, *p* <0.001 and R = -0.50,* p* <0.001, respectively). In children with haemophilia serum levels of Zn and phosphorus had a strong negative association with Haemo-QoL/Haem-A-QoL (R = -0.51, *p* = 0.01 and R = -0.51, *p* = 0.01, respectively), whereas, these associations were weak in adults (R = -0.28, *p* = 0.03 and R = -0.3, *p* = 0.02, respectively) (Figure 2). Moreover, serum level of Mg had a moderate negative association with Haemo-QoL/Haem-A-QoL in both children and adults with haemophilia (R = -0.45, *p* = 0.02 and R = -0.32,* p* = 0.01, respectively) (Figure 2). Results of the figure 2 indicates that serum Ca had no relation with Haemo-QoL/Haem-A-QoL in neither children nor adults (R = -0.28, *p* = 0.16 and R = -0.1, *p* = 0.52, respectively). 

**Association of serum vitamin D and assessed minerals with PedHAL/HAL**

**Figure 3:**
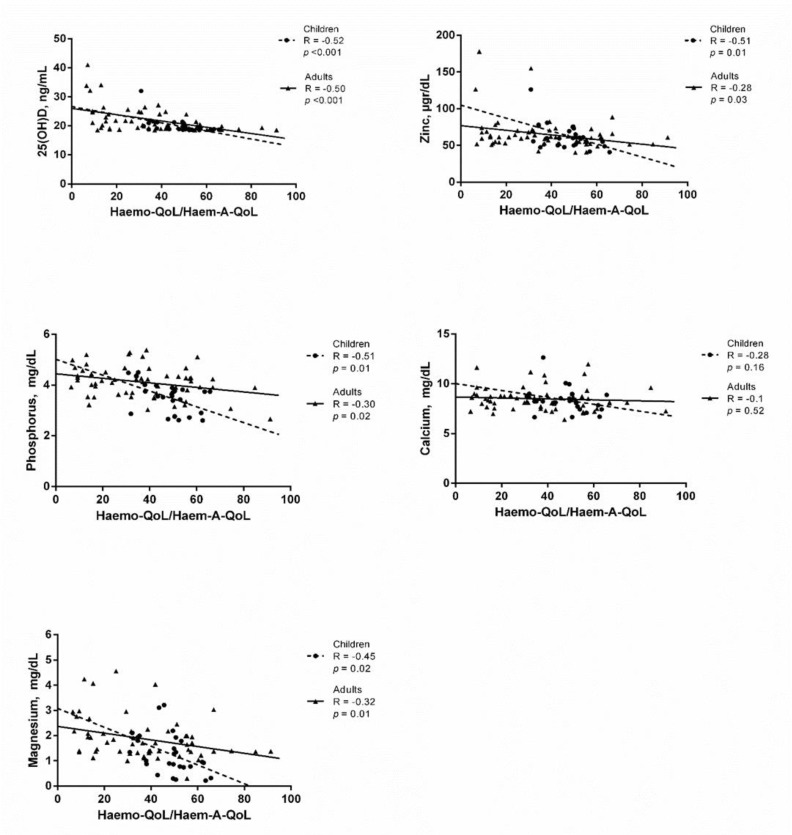
The association of the 25(OH)D and trace minerals with Haemo-QoL/Haem-A-QoL in patients with haemophilia. *P* values <0.05 were considered statistically significant.

As Figure 3 shows, blood 25(OH)D had a strong positive association with *PedHAL/HAL *in both children and adults (R = 0.69, *p* <0.001 and R = 0.65,* p* <0.001, respectively). Serum levels of Zn and Mg had a moderate positive association with PedHAL/HAL in both children and adults (Zn: R = 0.49, *p* = 0.01 and R = 0.45, *p* <0.001, respectively; Mg: R = 0.39, *p* = 0.04 and R = 0.36, *p* = 0.01, respectively). Serum levels of phosphorus had a strong positive association with PedHAL/HAL in children (R = 0.54, *p* <0.001), whereas, this association were moderate in adults (R = 0.36, *p* <0.001) (Figure 2). Serum Ca had no relation with PedHAL/HAL in neither children nor adults (R = 0.23, *p* = 0.24 and R = 0.14, *p* = 0.28, respectively). 

**Association of serum vitamin D and assessed minerals with HJHS**

**Figure 4:**
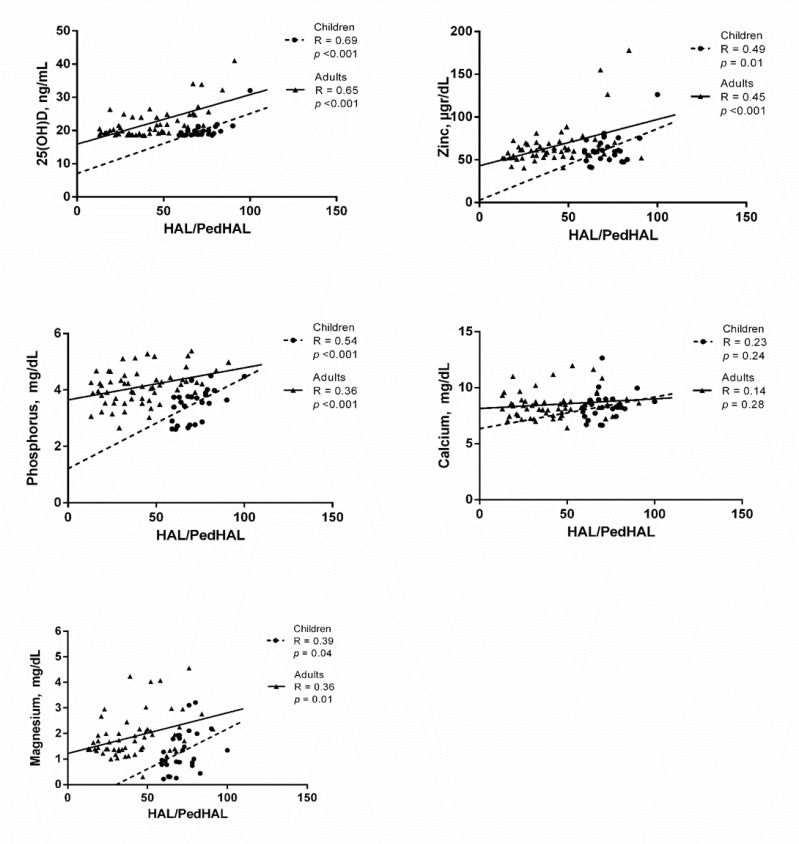
The association of the 25(OH)D and trace minerals with PedHAL/HAL in patients with haemophilia. *P* values <0.05 were considered statistically significant.

Circulating level of 25(OH)D had a strong negative association with HJHS in both children and adults (R = -0.51, *p* = 0.01 and R = -0.54,* p* <0.001, respectively) (Figure 4). As Figure 4 shows, serum concentrations of Zn and Mg had a moderate negative association with HJHS in both children and adults (Zn: R = -0.45, *p* = 0.02 and R = -0.41, *p* <0.001, respectively; Mg: R = -0.40, *p* = 0.04 and R = -0.32, *p* = 0.01, respectively). Serum levels of phosphorus had a strong negative association with HJHS in children (R = -0.60, *p* <0.001), whereas, this association were moderate in adults (R = -0.34, *p* =0.01) (Figure 2). Serum Ca had no relation with HJHS in neither children nor adults (R = -0.21, *p* = 0.29 and R = -0.06, *p* = 0.68, respectively). 

PedHAL/HAL, Pediatric Haemophilia/Haemophilia Activities List.

**Figure 5:**
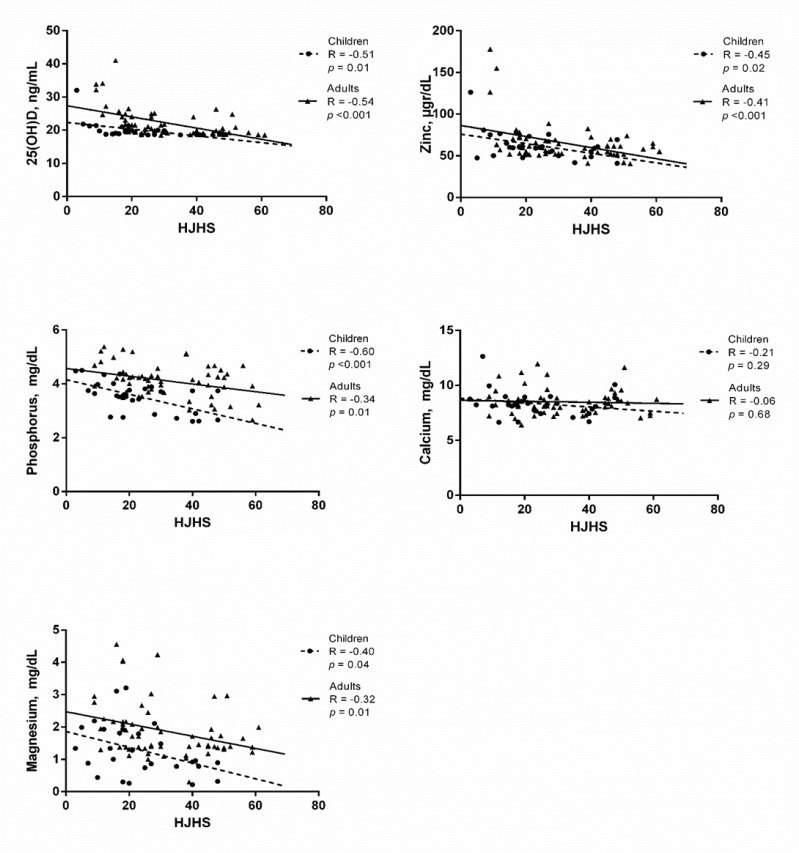
The association of the 25(OH)D and trace minerals with HJHS in patients with haemophilia. *P* values <0.05 were considered statistically significant.

HJHS, Haemophilia Joint Health Score.

**Table 4:** Association of serum biochemical factors with hemophilia-specific quality of life index (Haemo-QoL and Haem-A-QoL questionnaires)

Variables	Beta Coefficient									
	25(OH)D	*p* value	Zinc	*p* value	Phosphorus	*p* value	Calcium	*p* value	Magnesium	*p* value
Physical health	-0.47	<0.001	-0.3	<0.01	-0.27	0.01	-0.05	0.64	-.343	<0.001
Feelings	-0.43	<0.001	-0.32	<0.01	-0.17	0.11	-0.04	0.68	-.293	<0.01
View	-0.41	<0.001	-0.22	0.04	-0.08	0.42	-0.1	0.38	-.259	0.02
Family	-0.07	0.72	-0.11	0.59	-0.21	0.31	-0.26	0.19	-.301	0.13
Friend	-0.23	0.25	-0.28	0.17	-0.3	0.14	-0.32	0.11	-.430	0.03
Perceived support	-0.17	0.45	-0.16	0.48	-0.17	0.45	-0.31	0.17	-.320	0.15
Others	-0.32	0.11	-0.2	0.34	-0.15	0.46	-0.49	0.01	-.352	0.08
Sports and school	-0.47	<0.001	-0.3	<0.01	-0.26	0.01	-0.07	0.5	-.410	<0.001
Dealing	-0.25	0.02	-0.24	0.03	-0.06	0.6	-0.06	0.61	-.338	<0.001
Treatment	-0.41	<0.001	-0.25	0.02	-0.26	0.01	-0.14	0.21	-.382	<0.001
Future	-0.44	<0.001	-0.3	<0.01	-0.13	0.26	-0.11	0.38	-.413	<0.001
Relationship/partnership	-0.39	<0.001	-0.22	0.06	-0.06	0.63	-0.16	0.17	-.269	0.02
Work/school	-0.44	<0.001	-0.3	0.02	-0.13	0.31	0.07	0.58	-.307	0.02
Family planning	-0.43	<0.001	-0.22	0.09	0.02	0.85	-0.02	0.85	-.250	0.06
Total	-0.48	<0.001	-0.31	<0.01	-.219	0.04	-0.1	0.37	-.415	<0.001

## Discussion

 Results of the present study indicated that the serum levels of Zn, phosphorus and Mg were significantly (*p* <0.05) lower in HP as compared to HCs. In addition, serum ALP as a bone metabolism related marker was higher in HP. Furthermore, the percentage of patients who were vitamin D deficient (25(OH)D < 20 ng/ml) was higher in hemophilic group compared with healthy group (57.6% vs. 35.3%, respectively). 

Presence of the osteoporosis in HP is an important problem^12^. According to the previous studies the risk of osteoporosis related fractures in these patients is increasing^12^. The most important causes of osteoporosis in HP are hemorrhages into the musculoskeletal system, lack of adequate exercise due to the fear of bleeding, and vitamin D deficiency/insufficiency^12^. There is scarce data in the literature addressing the role of vitamin D in patients with haemophilia. Most of the previous studies reported a high prevalence of vitamin D deficiency in these patients especially in children^12^. In the study of Gerstner et al^6^ the prevalence of vitamin D deficiency (<20 ng/ml) in adults with haemophilia was 27%, and 70% of patients had decreased BMD. In 2014, Albayrak *et al., *reported that the prevalence of vitamin D deficiency in children with haemophilia was 68%^20^. In our study, 48.3% of adults with haemophilia were vitamin D deficient, and in children this rate was higher (77.8%). In addition, we found that the mean serum levels of 25(OH)D was statistically (*p* < 0.05) lower only in children with haemophilia as compared to age and sex matched healthy controls, whereas, there was no significant difference between hemophilic and healthy adults regarding the 25(OH)D. Similarly, the study of Gallacher *et al.* demonstrated that the serum levels of vitamin D was not different between hemophilic and healthy adults.^        14 ^ Another study in 2010 reported no difference in serum 25(OH)D between hemophilic and healthy adults^21^. Similar to our results, a study which investigated the serum status of vitamin D in children with haemophilia demonstrated that serum 25(OH)D was significantly lower in hemophilic children as compared with sex and aged matched healthy children^22^. Another study indicated that serum concentrations of 25(OH)D was significantly lower in children with haemophilia compared with healthy subjects^23^. Higher prevalence of vitamin D deficiency in hemophilic children could be due to the reduced physical activities and a tendency to a sedentary lifestyle in children with haemophilia^        24 ^. Families protect hemophiliacs more than other children due to the fear of joint damage and bleeding,^   25 ^ and this behavior may limit the physical activities which subsequently lead to reduced duration of children’s exposure to sunlight which is the main source of vitamin D.

In addition to the vitamin D, serum concentration of Zn and Mg are essential for the bone metabolism and the synthesis of bone matrix constituents^11^. Furthermore, serum abnormalities of Ca, phosphorus, PTH and ALP can represent the metabolic bone disorders^        26 ^. There is a limited data in the literature regarding the serum levels of trace minerals in HP. Most of the previous studies have reported the abnormalities of the serum levels of these factors^   16 ^. A study by Ghaniema *et al.* found that the serum levels of Zn and Mg were lower in hemophilic patients with age range of 7-40 years as compared to age and sex matched HCs^   16 ^. This was in accordance with our findings regarding the serum concentrations of Zn and Mg. It is of note that when comparing children and adults separately, circulating level of Mg was statistically lower (*p* = 0.028) only in hemophilic children and not in adults, and Zn was statistically lower (*p* <0.05) in both adults and children with haemophilia. However, in 2011, a study reported no significant difference regarding serum Mg levels between hemophilic children and HCs^22^. Furthermore, we found that serum level of phosphorus was significantly lower and ALP was higher in both children and adults with haemophilia, while there was no significant difference regarding serum Ca and PTH in neither hemophilic children nor adults compared with HCs. This is in line with the results of a previous study, which demonstrated that serum levels of ALP were higher in adults with severe haemophilia A, and there was no significant difference between groups regarding Ca and PTH^        14 ^. Results of another study indicated that serum level of ALP was significantly higher in hemophilic adults^21^. However, in the study of forty-four severe hemophilic children and 40 age and sex-matched HCs, no significant difference was found between groups regarding serum levels of Ca and phosphorus, whilst, serum level of PTH was increased in hemophilic children^22^. Although there are scarce data in the literature and the results of previous studies are controversial, most of these studies have come to the conclusion that in most of the HP, abnormalities of serum minerals and bone metabolism related factors are evident and most of these patients are at risk of low BMD and osteoporosis. 

In addition, previous studies found that serum minerals and vitamin D had a relationship with joint health and functional status of HP. In the study of Ghaniema *et al.* serum concentration of Zn had a significant negative correlation with the disorders of joints and the severity of functional disability in patients with haemophilia A, however, Mg had no correlation with these variables^   16 ^. Furthermore, in the study of Alioglu *et al.* serum 25(OH)D had a significant invers association with total joint score in HP^22^. Results of our study demonstrated that serum 25(OH)D, Zn, Mg and phosphorus had a significant negative association with joint impairment, and a significant positive association with functional activity. Of note, these relations were stronger in children with haemophilia. It is well documented that the serum trace minerals have roles as metalloenzymes in the synthesis of collagen and proteins which are involves in bone and joint structure, thus, are very important in maintaining bone and joint health^   27 ^. Moreover, several studies have revealed the beneficial roles of vitamin D in bone and joint health^        28 ^. It should be noted that the highest bone matrix mineralization occurs in childhood and adolescence, therefore, children’s skeletal system is very sensitive to deficiencies or insufficiencies of these minerals and vitamin D^   29 ^. Deficiencies or lower serum concentrations of these nutrients can affect the BMD^   29 ^. Previous studies demonstrated an association between BMD, and both the joint health score and the level of physical activity in hemophilic subjects^22^. 

Also, we found that higher serum levels of 25(OH)D, Zn, Mg and phosphorus had a significant association with better QoL in these patients. Results of the present study demonstrated that these associations were stronger in children than adults. To the best of our knowledge, our study is the first study which assessed the relation of serum vitamin D and minerals with QoL of HP. Previous studies indicated that vitamin D status are very important in the pathophysiology of psychological disorders^30^. Lower serum vitamin D was observed in patients with severe depression and anxiety^30^. In addition, a lower serum level of Zn, Mg and other minerals was reported in psychological illnesses^31^^,^^32^. These psychological disorders have important roles in impairment of QoL^33^. Moreover, recent studies have indicated a relation between higher serum vitamin D and higher QoL in different chronic diseases^34^. Therefore, association of serum vitamin D and minerals with QoL in HP can be explained through the roles of these nutrients in the pathophysiology of psychological disorders and subsequently in QoL. As a limitation, we did not evaluate other psychological factors in these patients, which, if measured, could increase our knowledge about the association of these nutrients with psychological factors in HP. However, the key advantage of the work is that it pertains to a population for which very little information exists on circulating vitamin D and minerals in HP. Another important strength of our study is a higher sample size in comparison to previous studies. Furthermore, for the first time we assessed the possible relation of vitamin D and minerals with QoL of HP. 

In conclusion, the present study indicated that, serum levels of Zn, phosphorus and Mg were lower in HP than healthy subjects. Results of our study demonstrated that the percentage of subjects who were vitamin D deficient was higher in HP as compared with healthy age and sex matched subjects, and also this rate was higher in children with haemophilia compared with adults. It is noteworthy that, mean serum level of 25(OH)D was lower only in children with haemophilia and not in adults when compared with HCs. Furthermore, we found that the lower serum concentrations of minerals and vitamin D had associations with lower physical activity, poor QoL and worst joint health in HP and these associations were stronger in children. The possibility of lower serum minerals and vitamin D in HP may be overlooked by hematologists due to their focus on matters such as bleeding and use of factor. In these patients, serum levels of vitamin D and minerals are not routinely measured, thus we suggest that all the HP especially children with severe haemophilia should be routinely screened in terms of serum vitamin D and minerals, however further studies are needed. Furthermore, large-scale studies are needed to investigate the effects of vitamin D and trace minerals supplementation on QoL and both joint and bon health in HP.
